# Continual Learning with Deep Neural Networks in Physiological Signal Data: A Survey

**DOI:** 10.3390/healthcare12020155

**Published:** 2024-01-09

**Authors:** Ao Li, Huayu Li, Geng Yuan

**Affiliations:** 1 Electrical and Computer Engineering, The University of Arizona, Tucson, AZ 85721, USA; hl459@arizona.edu; 2 BIO5 Institute, The University of Arizona, Tucson, AZ 85721, USA; 3 School of Computing, University of Georgia, Athens, GA 30602, USA; geng.yuan@uga.edu

**Keywords:** continual learning, deep learning, machine learning, physiological data, smart healthcare

## Abstract

Deep-learning algorithms hold promise in processing physiological signal data, including electrocardiograms (ECGs) and electroencephalograms (EEGs). However, healthcare often requires long-term monitoring, posing a challenge to traditional deep-learning models. These models are generally trained once and then deployed, which limits their ability to adapt to the dynamic and evolving nature of healthcare scenarios. Continual learning—known for its adaptive learning capabilities over time—offers a promising solution to these challenges. However, there remains an absence of consolidated literature, which reviews the techniques, applications, and challenges of continual learning specific to physiological signal analysis, as well as its future directions. Bridging this gap, our review seeks to provide an overview of the prevailing techniques and their implications for smart healthcare. We delineate the evolution from traditional approaches to the paradigms of continual learning. We aim to offer insights into the challenges faced and outline potential paths forward. Our discussion emphasizes the need for benchmarks, adaptability, computational efficiency, and user-centric design in the development of future healthcare systems.

## 1. Introduction

Physiological signals reflect the biological activities, which can be measured within the human body [1]. These include electrocardiograms (ECG), electroencephalograms (EEG), electromyograms (EMG), blood pressure signal, respiratory signal, and oxygen saturation (SpO_2_). These signals are crucial for medical diagnostics and health monitoring. Advances in wearable technology and the internet of medical things (IoMT) have facilitated the continuous capture and analysis of such signals, significantly improving the timeliness and precision of healthcare interventions. With the rapid progression of medical technology, deep-learning algorithms are increasingly employed to process this stream of data, offering unprecedented efficiency and predictive power in diagnostics and monitoring. Recent studies have highlighted deep learning’s impressive ability to uncover intricate patterns within high-dimensional physiological signal data, indicating a transformative potential in healthcare applications [2,3,4].

**Figure 1:**
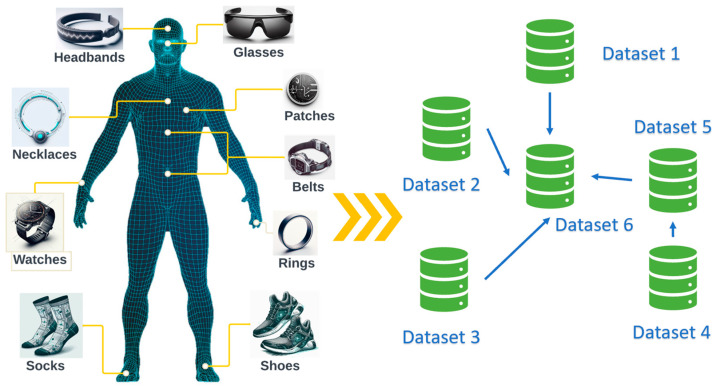
Wearable sensors and evolving datasets.

While deep learning has shown significant promise, traditional approaches often struggle with the dynamic and evolving nature of physiological data. Figure 1 provides a graphical representation of the variety of wearable sensors available and the continually evolving datasets they generate. As wearable sensors continuously accumulate data, they capture a spectrum of physiological parameters and health indicators. Unfortunately, the dynamic nature of these data often challenges traditional deep-learning approaches, which are typically trained once and then deployed [5].

Distinct from traditional deep-learning approaches, continual-learning—also known as lifelong-learning—techniques are designed to handle evolving datasets, adapting to streaming data and changing environments [6,7]. They pivot as the environments change and are thus ideally suited for shifting patient populations and new data paradigms. In the era of smart healthcare, where patient care is becoming increasingly personalized and data-driven, the ability of models to adapt over time without forgetting previously learned information is paramount. While previous review papers have delved into continual learning in domains such as computer vision, robotics, and natural language processing [7,8,9,10,11], and others have explored deep learning in physiological signals [3,12], there remains an absence of a unified, comprehensive review focused specifically on continual learning in physiological signal analysis. This absence represents a significant gap; without such consolidated resource, the potential for fragmented research and duplicated efforts increases, hindering the pace of innovation. Recognizing this crucial need, we aim to bridge this knowledge gap. We endeavor to present an overview of the current techniques, applications, challenges, and the future of continual learning in physiological signal analysis.

## 2. Continual Learning

### 2.1. From Traditional Approaches to Continual Learning

In the realm of smart healthcare, the transition from traditional to continual learning represents a significant paradigm shift. While deep-learning techniques have gained traction in healthcare applications [12,13], the constraints of traditional deep neural networks are becoming more evident. The traditional models—predominantly trained in a batch setting—excel with static datasets, but their performance suffers when adapting to dynamic data streams [14]. This phenomenon is called catastrophic forgetting. Continual learning, on the other hand, champions an iterative learning approach. Here, the models continually update their knowledge base with incoming data, designed specifically to embrace new information without erasing prior knowledge. In the dynamic environment of smart healthcare, this ability to incorporate and adapt to fresh data can greatly enhance diagnostic precision and pave the way for tailored treatment plans. Consequently, there is a mounting interest in employing continual learning for physiological signal analysis.

Several fundamental concepts emerge as pivotal in continual learning. The delicate balance of adapting to new information while retaining previously acquired knowledge—referred to as the plasticity–stability dilemma—is a critical challenge in continual learning [11]. Additionally, the ability of models to transfer knowledge from past experiences to new related tasks plays an essential role in enhancing their effectiveness. Furthermore, domain adaptation is a key, which enables models to adapt to new data domains without discarding valuable insights from older data. These principles are instrumental in developing robust and adaptable continual-learning models tailored to healthcare’s dynamic and evolving demands.

**Figure 2:**
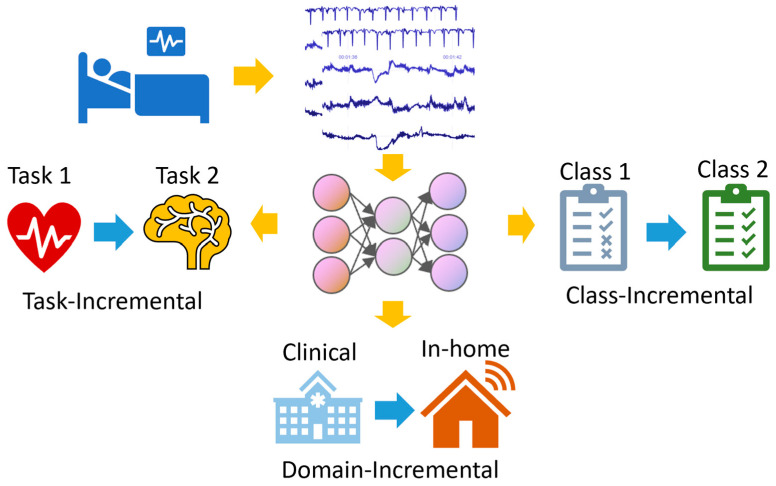
A diagram illustrating the three scenarios of continual learning.

Continual learning can be segmented into three primary scenarios, as depicted in Figure 2. Task-Incremental Learning: In this scenario, the model incrementally learns a set of clearly distinguishable tasks. For example, a monitoring system might first analyze ECG signals for arrhythmia detection and later expand to score sleep stages. Domain-Incremental Learning: This scenario involves training the model for the same task but across varied situations. Consider a model optimized for ECG data from a clinical setting, which is later adapted to interpret data from wearable devices. Class-Incremental Learning: In this scenario, the model can learn to classify an increasing number of classes. For instance, a system initially trained to distinguish between normal and abnormal heartbeat may later be updated to identify specific types of arrhythmias. When these scenarios are combined, the challenges intensify. Well-crafted continual-learning strategies are required to adeptly manage these varied scenarios.

Evaluating the performance of continual-learning models requires specific metrics and benchmarks. While traditional metrics retain their relevance, the unique nature of continual learning necessitates more specialized metrics [15]. Backward transfer is a commonly used metric, which evaluates how effectively a model retains its performance on previous tasks while acquiring new knowledge. Additionally, the integration of benchmark datasets specifically tailored for continual learning, such as sequential versions of static datasets, offers invaluable insights. These instruments provide a more precise understanding of the model’s ability to learn continually and adaptively.

### 2.2. Continual-Learning Approaches

Research in continual learning has led to the development of various techniques for incremental learning from data streams. These techniques can be grouped into replay-based, regularization-based, and architecture-based approaches.

#### 2.2.1. Replay-Based Approaches

Replay-based approaches tackle the problem of catastrophic forgetting by retaining a small sub-set of old data to “replay” during the training for new tasks. For example, a model analyzing ECG signals could store a representative sample of past ECG signals and incorporate these into the learning process when adapting to new tasks. This replay of historical data allows the model to refresh its memory, thereby maintaining performance in older tasks while adapting to new ones.

Replay-based approaches offer a variety of techniques. Rebuffi et al. [16] introduced iCaRL, which is a class-incremental learner that performs classification using the nearest exemplar algorithm and prevents catastrophic forgetting by using episodic memory. Another noteworthy contribution is gradient episodic memory (GEM) for task-incremental learning, which leverages episodic memory to store a sub-set of observed examples from previous tasks [17]. Prioritized experience replay enhances experience replay by not treating all samples equally [18]. Instead, samples yielding higher learning potential—often measured by the temporal difference error—are sampled more frequently. This selective replay can lead to more efficient and effective learning. Recently, Arani et al. [19] introduced a novel complementary learning system with dual memory experience replay (CLS-ER), which maintains short-term and long-term semantic memories, which interact with episodic memory. This method achieved state-of-the-art performance across both standard and more complex continual-learning benchmarks. On the other hand, generative experience replay—exemplified by deep generative replay [20]—employs generative models to synthesize new data samples, which are representative of past tasks. This not only reduces the need for large memory storage but also allows for the generation of more diverse training samples [21].

While these methods offer significant advantages—including flexibility in integration with various neural network architectures and adaptability to different learning scenarios—they are not without limitations. The quality of the replay data is crucial. If the stored or generated experiences are not representative or are poorly chosen, they can introduce biases or fail to capture the complexity of past tasks, thereby affecting the model’s overall performance.

#### 2.2.2. Regularization-Based Approaches

Regularization-based approaches are particularly useful for physiological data, where the underlying physiological mechanisms may share common features across different tasks. By imposing regularization constraints, the model can learn to identify and preserve these common features, facilitating better generalization across tasks.

One of the early pioneers in this domain is the learning-without-forgetting (LwF) algorithm introduced in 2016. LwF leverages knowledge distillation techniques to regularize the model during the training phase on new tasks, thereby mitigating the loss of previously acquired knowledge [22]. In the subsequent year, elastic weight consolidation (EWC) emerged as a seminal work, introducing a regularization term into the loss function to penalize alterations to crucial weights identified during prior training sessions [23]. Recently, dual regularization techniques employing a memory set were introduced, which are particularly effective in domain-incremental learning [24]. Moreover, geometric regularization of class representations was introduced as a simplistic yet potent tool for continual learning [25].

The regularization-based methods in continual learning come with their own set of advantages and limitations. One of the primary advantages is their simplicity and computational efficiency. These methods often involve adding a regularization term to the loss function, which is computationally less demanding compared to methods, which require architectural modifications or storing large amounts of data. This makes them particularly suitable for scenarios where computational resources are limited. However, the limitations are also noteworthy. One limitation is the potential for hyperparameter sensitivity; the effectiveness of the regularization term often depends on the choice of hyperparameters, which may require extensive tuning. Additionally, while these methods are effective in mitigating catastrophic forgetting to some extent, they are not always capable of eliminating it, especially in scenarios where tasks are highly dissimilar or when the number of tasks is large.

#### 2.2.3. Architecture-Based Approaches

Architecture-based approaches employ specialized neural network architectures to partition the model into different components, each responsible for learning specific tasks or features. This modular approach allows for greater flexibility in updating individual tasks while minimizing interference with others.

One of the early studies in this domain included progressive neural networks (PNN) [26], introduced to extend the architecture by allocating a new sub-network for each task, thereby preserving the knowledge of previous tasks while accommodating new ones. Aljundi et al. [27] introduced the expert gate, consisting of a set of gating autoencoders designed to learn representations for the current task. During the test, these autoencoders automatically forward the test sample to the relevant network. In the subsequent years, efficient architecture search for continual learning (CLEAS) was developed, focusing on optimizing the architecture itself for continual-learning scenarios [28]. Graffieti et al. [29] also introduced a novel hybrid approach, which combines architectural elements and replay memory management for effective continual learning. This approach is effective in several scenarios, including temporally ordered streams as input data, strong correlation of samples in short time ranges, high data distribution drift over a long time frame, and heavy class unbalancing. Another study further emphasized the significant impact of architecture choice on continual-learning performance [30]. Mirzadeh et al. [30] found that different neural network architectures have different learning and retention capabilities and that slight changes in the architecture can result in significant changes in performance in a continual-learning setting.

Architecture-based approaches offer several advantages, including their inherent ability to mitigate catastrophic forgetting through their design and their flexibility in adapting to a wide range of tasks without requiring extensive retraining. These methods can also be computationally efficient, as they often focus on optimizing the architecture itself. However, they come with limitations, such as the complexity of design and implementation and potential scalability issues, which indicate that there is still much to explore in terms of optimizing these architectures for various continual-learning scenarios.

## 3. Reviews

We conducted a literature search on Google Scholar, PubMed, and IEEE Xplore using combinations of the terms “Continual Learning”, “Lifelong Learning”, “Incremental Learning”, “ECG”, “EEG”, “EMG”, and “Physiological Signal”. The selection criteria for articles found through this search strategy were as follows: (1) The study utilizes at least one physiological signal; (2) The study encompasses one of the primary scenarios, which include task-incremental learning, class-incremental learning, or domain-incremental learning; (3) The study employs a deep neural network; (4) The study employs one or more continual-learning approaches, including replay-based approaches, regularization-based approaches, and architecture-based approaches; (5) The study is focused on healthcare; (6) The study is peer-reviewed and published between January 2021 and September 2023. Based on these criteria, we identified eight articles focusing on the application of continual-learning methods to physiological signals. A summary of these articles is provided in Table 1.

### 3.1. Continual Learning for ECG Signals

Ammour et al. [31] introduce a learning-without-forgetting approach for heartbeat classification. When a new task is introduced, the network expands with a new classification layer, which uses a Softmax activation function. This new layer learns the classes of the new task. During training for this new task, both the shared layers and the output layers of previous tasks are fine-tuned using pseudo-labels, aiding in knowledge retention. The task selector then stores feature prototypes for each task. Using a distance matching network, it determines the most suitable task to classify a new test sample. This method has been validated in three open-access datasets: MIT-BIH Arrhythmia Database [32], St Petersburg INCART 12-Lead Arrhythmia Database [33], and MIT-BIH Supraventricular Arrhythmia Database [34]. However, there are potential scalability issues; as new tasks are added, the model becomes more complex.

Kiyasseh et al. [35] proposed a replay-based approach—termed CLOPS—for cardiac arrhythmia classification. CLOPS employs an importance-guided buffer storage and an uncertainty-based buffer acquisition mechanism. The method was tested in both class-incremental and domain-incremental scenarios across four datasets: iRhythm Cardiology Dataset [36], Chapman University and Shaoxing People’s Hospital Dataset [37], PhysioNet 2017 Challenge Dataset [38], and PhysioNet 2020 Challenge Dataset [39]. CLOPS outperformed earlier algorithms. However, the approach assumes a portion of training data can be stored temporarily in a buffer for future use, which may raise patient privacy and data storage concerns. Furthermore, CLOPS was only tested on single-lead ECG data.

Sun et al. [40] proposed a meta self-attention prototype incrementor (MAPIC) framework for few-shot class-incremental learning in medical time series classification. The MAPIC framework was tested on the MIT-BIH Arrhythmia Database [32,33], MIT-BIH Long-Term ECG [33], FaceAll (facial outline) [41], and UWave (Gesture) Datasets [41], demonstrating top-tier performance. However, the framework was only evaluated on smaller datasets, indicating the need for further assessment before deployment in smart healthcare settings.

Gao et al. [42] introduced a parameter-isolation-based ECG continual-learning (ECG-CL) approach, which emphasizes leveraging both local morphological and global rhythm information in ECG interpretation. The method was validated across four large open-access datasets, including China Physiological Signal Challenge 2019 (CPSC 2019) [43], 12-Lead QRS [44], ICBEB 2018 [45], and PTBXL [46], showcasing its adaptability to diverse learning schemes, such as domain-incremental learning and class-incremental learning. Nevertheless, its efficacy depends on the availability and quality of labeled data. In scenarios with sparse or inconsistent data, its applicability might be restricted. To address this, the authors recommend incorporating unsupervised learning techniques to boost the model’s resilience and adaptability.

### 3.2. Continual Learning for Other Physiological Signals

Hua et al. [47] introduced a task-incremental framework for gesture classification. The study presented an early and late fusion convolutional neural network (ELFCNN) architecture, leveraging the frequency spectrum. By integrating ELFCNN with the hybrid data over-/down-sampling (HDOD) technique, the research showcased the potential of incremental learning in sEMG-based gesture classification. The tests indicated a marked improvement in classification accuracy, rising from 0.47% to 0.71%, compared with other prevalent incremental learning methods. However, this algorithm was tested solely on the Ninapro DB2 dataset [48].

Armstrong and Clifton [5] assessed an array of continual-learning techniques on two substantial ICU datasets—eICU and MIMIC-III [49,50]—specifically in domain-incremental scenarios. Their findings revealed that domain shift continues to pose challenges across an extensive series of tasks. Only replay-based methods displayed consistent long-term performance.

Sun et al. [51] presented MetaCL, a framework combining federated learning and blockchain, optimized for physiological signal classification on the IoMT. MetaCL responds to the dynamic attributes of physiological signals by using a shared feature extractor, which harnesses horizontal federated learning to reduce data leakage. The system also integrates a knowledge base, updated through a split-based method, effectively preventing catastrophic forgetting in machine-learning models. A micro-classifier module, utilizing mean-based model transfer and Kullback–Leibler divergence regularization, adjusts to new classes and ambiguous boundaries. MetaCL was validated across four open-access datasets, including MIT-BIH Arrhythmia Database [32], Wrist PPG During Exercise [52], St Petersburg INCART 12-Lead Arrhythmia Database [33], and Sleep-EDF Expanded [53]. MetaCL showed good robustness in different continual-learning scenarios. However, the framework’s reliance on federated learning for feature extractor training and the use of blockchain for privacy protection could lead to higher computational demands.

Sun et al. [54] also presented PCDOL, an algorithm designed for online continual learning in sensor time series classification, specifically for nanorobots. PCDOL utilizes a temporal convolutional network (TCN) for feature extraction and integrates a multi-component loss function to combat catastrophic drift. Five datasets were employed in this study, including MIT-BIH Arrhythmia Database [32], MIT-BIH Supraventricular Arrhythmia [34], St Petersburg INCART 12-Lead Arrhythmia [33], MIT-BIH Long-Term ECG [33], and Wrist PPG During Exercise [52]. The algorithm underwent testing on ECG and PPG data streams, divided into tasks with diverse categories. Compared to the existing methods, PCDOL not only showed superior task-specific accuracy but also exhibited strong continual-learning performance. However, the effectiveness of the algorithm depends on the precise tuning of its loss term, which might pose challenges in situations demanding quick adaptation.

**Table 1:** Summary of works using continual-learning approaches with physiological signals.

Authors	Objectives	Signals	Datasets	Incremental Learning Scenarios
Ammour et al. [31]	A learning-without-forgetting approach for ECG heartbeat classification	ECG	MIT-BIH Arrhythmia Database, St Petersburg INCART 12-Lead Arrhythmia Database, MIT-BIH Supraventricular Arrhythmia Database	Task-incremental
Kiyasseh et al. [35]	A replay-based approach (CLOPS) for cardiac arrhythmia classification	ECG	Cardiology Dataset, Chapman Dataset, PhysioNet 2017 Challenge Dataset, PhysioNet 2020 Challenge Dataset	Class-incremental Domain-incremental
Sun et al. [40]	A meta self-attention prototype incrementor framework for medical time series classification	ECG	MIT-BIH Arrhythmia Database, MIT-BIH Long-Term ECG, FaceAll, UWave	Class-incremental
Gao et al. [42]	A parameter-isolation-based ECG continual-learning (ECG-CL) approach	ECG	CPSC 2019, 12-Lead QRS, ICBEB 2018, PTBXL	Class-incremental Domain-incremental Task-incremental
Hua et al. [47]	A framework with ELFCNN and HDOD for gesture classification	EMG	Ninapro DB2	Task-incremental
Armstrong et al. [5]	Evaluation of a variety of continual-learning methods on longitudinal ICU data	Multivariate time series	eICU-CRD, MIMIC-III	Domain-incremental
Sun et al. [51]	A federated learning and blockchain framework tailored for physiological signal classification	EEG, ECG, PPG	MIT-BIH Arrhythmia Database, Wrist PPG During Exercise, St Petersburg INCART 12- Lead Arrhythmia, Sleep-EDF Expanded	Task-incremental, Domain-incremental
Sun et al. [54]	An algorithm for online continual learning in sensor time series classification within the context of nanorobots	ECG, PPG	MIT-BIH Arrhythmia Database, MIT-BIH Supraventricular Arrhythmia, St Petersburg INCART 12- Lead Arrhythmia, MIT-BIH Long-Term ECG, and Wrist PPG During Exercise	Class-incremental

### 3.3. Continual-Learning Datasets

In the field of physiological signal analysis, a variety of benchmark datasets have played a pivotal role in research and development. Among them, the MIT-BIH Arrhythmia Database stands out with ECG recordings from 47 subjects [32], setting a benchmark for arrhythmia detection algorithms. The MIT-BIH Supraventricular Arrhythmia Database augments this collection with 78 half-hour ECG recordings [34], focusing on supraventricular arrhythmias. The St Petersburg INCART 12-Lead Arrhythmia Database complements this with 75 half-hour extracts from 32 Holter records [33], targeting coronary artery disease. These datasets, along with PTBXL [46], 12-Lead QRS [44], and iRhythm Cardiology Dataset [36], provide well-annotated ECG signals for a broad spectrum of cardiac conditions. The Chapman datasets [37], together with the PhysioNet 2017 [38], 2020 Challenge Datasets [39], CPSC 2019 [43], and ICBEB 2018 [45], contribute extensive ECG data crucial for pushing the boundaries of algorithmic design in this domain.

Transitioning from cardiac-focused collections, datasets such as Sleep-EDF Expanded offer comprehensive polysomnography data [53], including EEG, EOG, chin EMG, and event markers from 197 whole-night records. For applications in prosthetics and gesture recognition, the Ninapro DB2 database provides surface EMG signals, inertial, kinematic, and force data from 40 individuals performing various hand movements [48]. In the critical care field, the eICU-CRD and MIMIC-III datasets offer extensive ICU data, from vital signs to detailed clinical notes [49,50]. Datasets such as Wrist PPG During Exercise are instrumental for developing algorithms, which can withstand motion artifacts and exercise-induced changes [52]. Spanning from tens to thousands of subjects and encompassing a multitude of signal types, these datasets are richly annotated by experts and provide invaluable resources for researchers to develop, validate, and test continual-learning models tailored for the dynamic landscape of smart healthcare.

## 4. Discussion, Challenges, and Future Exploration

The interest in continual learning has catalyzed the evolution of numerous methodologies specifically tailored to capacitate models in learning incrementally from physiological signal data. Colleagues have pioneered frameworks optimized for a diverse range of signals, including ECG, EEG, PPG, EMG, and vital signs. These frameworks not only testify to the dynamism of the field but also highlight the quest for versatile solutions, which cater to various physiological monitoring needs. A wide array of benchmarks was explored to rigorously evaluate and compare the performance of these proposed systems.

### 4.1. Benchmarks and Performance Assessment

As the domain of continual learning tailored to physiological signal data evolves, a challenge revolves around the adept implementation of continual learning across diverse healthcare paradigms, spanning from task-incremental to class-incremental and domain-incremental learning. In the context of integration of continual learning in modern healthcare, there is an ever growing need to ensure these solutions are compatible with and optimized for a range of healthcare applications. Most of the current models, though promising, are calibrated and validated predominantly using short-term and limited datasets. However, the inherent nature of healthcare frequently necessitates long-term monitoring. This underscores the imperative for extensive studies dedicated to amassing physiological signal data. Such data can simulate genuine real-world scenarios, serving as a test for the resilience and adaptability of continual-learning frameworks. Looking forward, as the complexity and variety of physiological data increase, there is an implicit need to develop benchmarks, which can more comprehensively capture the intricacies and nuances of real-world scenarios, paving the way for solutions, which are both robust and universally applicable.

Moreover, the assessment of continual-learning systems’ performance presents its own set of unique challenges and complexities. Traditional metrics—such as the area under the receiver operating characteristic curve and the F1 score—may fall short in capturing the dynamic nature of models, which evolve over time. It is imperative to develop metrics, which emphasize system adaptability, resilience against catastrophic forgetting, and computational efficiency. For instance, forward transfer provides insights into a model’s ability to utilize past knowledge when learning new information [15]. Conversely, backward transfer evaluates how the performance in a previously learned task improves by leveraging data from subsequent tasks [15]. However, these metrics have limitations, especially in capturing a model’s long-term adaptability and its generalization abilities across unseen tasks. Beyond simply performance, the ethical landscape of continual learning in healthcare is intricate. Ensuring fairness, stability, and the avoidance of biases across diverse patient populations becomes paramount. With patients and healthcare professionals at the heart of these technological advancements, there is a pressing need to design algorithms, which are transparent, ethical, and user-centric. The path ahead demands comprehensive investigations in order to refine and mature the metrics tailored for continual-learning scenarios, ensuring they align with both the technical and ethical demands of the healthcare domain.

### 4.2. Energy Efficiency and Computation Capability

The popularity of IoMT-based monitoring of physiological signals has increased in recent years due to its advantages, including enhanced patient mobility, continuous patient observation, and reduced healthcare expenses [55,56]. Wearable devices and edge computing—as the main carriers of IoMT—have opened doors to new possibilities in continual learning [57]. These devices, particularly wearables, generate a constant stream of time series data, which can be harnessed for real-time learning applications. With the consideration of data privacy and varying external environments [58], it would be a desirable solution to conduct continual-learning algorithms locally on wearable or edge devices.

However, there are several distinct challenges remaining. Wearable or edge devices do not usually have a consistent power supply (e.g., battery power); therefore, energy efficiency becomes a critical factor to ensure continuous functionality. Moreover, the limited computing resources and capabilities of these devices render it difficult to meet real-time requirements while accomplishing complex tasks. While neural network compression techniques—e.g., pruning [59,60] and quantization [61,62]—together with operating system level optimizations [63,64] are the potential solutions for cost-effective continual learning when using edge devices [65], it is important to note that the research to date has mainly focused on computer vision tasks.

The unique characteristics of physiological signals make them distinct from conventional computer vision tasks or natural language processing tasks. Recent literature made an initial attempt to apply sparse training algorithms to save the computing cost of training for time series data [66,67]. However, research on adopting and optimizing on-device continual learning for physiological signals is still largely absent and an active area worth further exploration. Additionally, as these wearable technologies become more ubiquitous in healthcare, understanding the perspectives and needs of the end users, including patients and healthcare professionals, will be vital for the broader acceptance and success of these systems.

### 4.3. Future Directions

For future research in continual learning within healthcare, a comprehensive approach is essential, focusing on both data and evaluation standards. The need for longitudinal and diversified datasets, which accurately reflect real-world healthcare scenarios, is paramount. These datasets are crucial not only for developing innovative continual-learning approaches but also for ensuring that these approaches are relevant and applicable in practical healthcare settings. Simultaneously, the advancement of the field necessitates the establishment of standardized benchmarks and evaluation metrics specifically designed for continual learning in healthcare. This crucial step requires a multi-disciplinary effort involving engineers, computer scientists, and healthcare professionals. Their collective expertise is needed to develop and validate benchmarks, which are technically sound and directly applicable to the dynamic environment of healthcare. These benchmarks should comprehensively assess models, going beyond mere accuracy to evaluate adaptability, resilience, and long-term performance in various healthcare scenarios.

In addressing the prevalent challenges of catastrophic forgetting and model generalization across different physiological conditions, future research must explore novel learning paradigms. These could be inspired by an inter-disciplinary blend of insights from machine learning, neuroscience, and cognitive science. Equally important is the need to balance model complexity with computational efficiency, especially in developing technologies for wearable devices. This might involve creating advanced model compression techniques specifically suited for time series physiological data.

By focusing on these critical areas—from data and evaluation standards to technological innovation—future research is poised to significantly advance deep-learning applications in healthcare. This progress is crucial for developing healthcare solutions, which are more personalized, accurate, and efficient, ultimately leading to a transformative impact on patient care and outcomes.

### 4.4. Limitations

While this review provides a comprehensive overview of the evolving landscape of continual learning within the context of physiological signals, there are notable limitations to be addressed. First, our review predominantly sourced from peer-reviewed English-language publications, potentially overlooking significant advancements, methodologies, or perspectives from non-English publications. This linguistic limitation could lead to a potential bias toward English-speaking research communities, inadvertently missing out on the global breadth of work in this domain. Second, our focus was primarily centered on deep neural networks when discussing continual-learning technologies. As a result, other machine-learning models, such as support vector machines, decision trees, or ensemble methods, which might also have applications in continual learning for physiological signals, were not explored in depth. This decision was made in order to maintain a sharp focus on the rapidly evolving area of deep learning; however, it is crucial to recognize that deep learning is only a sub-field of machine learning and artificial intelligence.

## 5. Conclusions

The interest in continual learning for physiological signal data is evident from the increasing number of publications in this area. However, the existing body of research does not fully encompass the vast potential healthcare applications, which could benefit from such techniques. This survey provides a comprehensive overview of the evolving landscape of continual learning within the context of physiological signals, shining light on its promising avenues, inherent challenges, and future exploration. While the path forward presents significant challenges—ranging from the development of tailored metrics to the computational limits of edge devices—these obstacles also offer opportunities for innovative breakthroughs. By tackling these intricate issues, there is potential to revolutionize healthcare systems, making them intelligent, adaptive, equitable, and resource-efficient.
